# Leveraging Personal Technologies in the Treatment of Schizophrenia Spectrum Disorders: Scoping Review

**DOI:** 10.2196/57150

**Published:** 2024-09-30

**Authors:** Jessica D'Arcey, John Torous, Toni-Rose Asuncion, Leah Tackaberry-Giddens, Aqsa Zahid, Mira Ishak, George Foussias, Sean Kidd

**Affiliations:** 1 Schizophrenia Division Centre for Addiction and Mental Health Toronto, ON Canada; 2 Clinical Psychological Sciences Department of Psychology University of Toronto Scarborough Toronto, ON Canada; 3 Department of Psychiatry Beth Israel Deaconess Medical Center Harvard Medical School Boston, MA United States; 4 Department of Psychology Research and Clinical Training Concordia University Montreal, QC Canada

**Keywords:** schizophrenia, digital mental health, personal technology, access to specialized resources, mental health, scoping review, mental health care, feasibility, efficacy, clinical integration, support, specialized care, care, database, schizophrenia spectrum disorder, text messaging, text, user feedback, usability, acceptability, satisfaction, engagement, digital health, digital mental health, technology, health technology, mood disorder, mood disorders, neurodevelopment, eHealth, mobile phone

## Abstract

**Background:**

Digital mental health is a rapidly growing field with an increasing evidence base due to its potential scalability and impacts on access to mental health care. Further, within underfunded service systems, leveraging personal technologies to deliver or support specialized service delivery has garnered attention as a feasible and cost-effective means of improving access. Digital health relevance has also improved as technology ownership in individuals with schizophrenia has improved and is comparable to that of the general population. However, less digital health research has been conducted in groups with schizophrenia spectrum disorders compared to other mental health conditions, and overall feasibility, efficacy, and clinical integration remain largely unknown.

**Objective:**

This review aims to describe the available literature investigating the use of personal technologies (ie, phone, computer, tablet, and wearables) to deliver or support specialized care for schizophrenia and examine opportunities and barriers to integrating this technology into care.

**Methods:**

Given the size of this review, we used scoping review methods. We searched 3 major databases with search teams related to schizophrenia spectrum disorders, various personal technologies, and intervention outcomes related to recovery. We included studies from the full spectrum of methodologies, from development papers to implementation trials. Methods and reporting follow the PRISMA (Preferred Reporting Items for Systematic Reviews and Meta-Analyses) guidelines.

**Results:**

This search resulted in 999 studies, which, through review by at least 2 reviewers, included 92 publications. Included studies were published from 2010 to 2023. Most studies examined multitechnology interventions (40/92, 43%) or smartphone apps (25/92, 27%), followed by SMS text messaging (16/92, 17%) and internet-based interventions (11/92, 12%). No studies used wearable technology on its own to deliver an intervention. Regarding the stage of research in the field, the largest number of publications were pilot studies (32/92, 35%), followed by randomized control trials (RCTs; 20/92, 22%), secondary analyses (16/92, 17%), RCT protocols (16/92, 17%), development papers (5/92, 5%), and nonrandomized or quasi-experimental trials (3/92, 3%). Most studies did not report on safety indices (55/92, 60%) or privacy precautions (64/92, 70%). Included studies tend to report consistent positive user feedback regarding the usability, acceptability, and satisfaction with technology; however, engagement metrics are highly variable and report mixed outcomes. Furthermore, efficacy at both the pilot and RCT levels report mixed findings on primary outcomes.

**Conclusions:**

Overall, the findings of this review highlight the discrepancy between the high levels of acceptability and usability of these digital interventions, mixed efficacy results, and difficulties with sustained engagement. The discussion highlights common patterns that may underscore this observation in the field; however, as this was a scoping review, a more in-depth systematic review or meta-analysis may be required to better understand the trends outlined in this review.

## Introduction

### Background

Research on digital mental health care in groups with serious mental illness (SMI), including schizophrenia spectrum disorders (SSDs), has grown slowly compared to other mental health conditions such as anxiety and depression [1,2]. Historically, this has been due to concerns about the ability to afford and use technology (ie, device ownership, access to the internet, and cellular data plans), understand the limitations of the technology (ie, privacy, crisis planning, and digital literacy), and make effective gains from digitally delivered content.

Recent research shows that cell phone and smartphone ownership and internet access are high in populations with SMI with and without psychosis [3,4] and have shown to be an adequate means of service delivery over the COVID-19 pandemic. Leveraging personal technologies (ie, laptops, tablets, smartphones, and wearable technologies) has also been framed as a critical measure to address access to specialized mental health care in remote areas and populations identified as marginalized, as access to smartphones and the internet is improving in these communities [5-7]. In addition, it has the potential to address barriers to stigma, time, and cost that limit the frequency and duration of available mental health care [8,9]. Given the potential to overcome such significant barriers, experts predict the use of personal technologies to deliver or assist with mental health care to continue to grow and hope to capitalize on the burst of enthusiasm brought about by the COVID-19 pandemic [10-12].

Although technologically delivered and assisted care is steadily gaining credibility and acceptability among mental health care professionals, there remain questions about the efficacy, safety and potential limitations of technologically delivered care in SSDs [13]. In fact, research has found that out of the publicly available technology advertised for mental health care, only 3% have scientific evidence supporting their use [14]. In schizophrenia and psychosis, a commercial review found that only 6 publicly available apps out of 700 were supported by scientific evidence, and many contained stigmatizing themes and misinformation [15].

To this end, it is critical to identify evidence-based digital mental health tools available for use on personal technologies by individuals with SSDs and evaluate the state of research in the field and readiness for implementation. Previous reviews in this area have tended to focus on 1 technical modality (eg, mobile apps [16] and SMS text messaging [17]) or 1 aspect of recovery (eg, relapse prevention [18]) but have yet to examine the field collectively across technological platforms, treatment targets, and therapeutic approaches to provide a high-level snapshot of the extant literature.

### This Study

This study is a scoping review examining the use of personal technologies to deliver digital mental health interventions in the treatment of SSDs. The overarching aim was to examine the state of the existing evidence supporting digital health tools for individuals with SSDs. Specifically, we identified gaps in the literature and explored the next steps for future research as the field moves toward implementation. To this end, we examined studies across the research stage, from development papers to implementation studies, using various types of commonly owned personal technologies that may be used to support clinical outcomes and recovery in populations experiencing SSDs. Personal technologies were chosen as the target technology for this review, as it can be argued that this may be the most efficient way to increase access to specialized information and care for individuals who cannot access traditional care consistently. It is also plausible that personal technologies may be the most accessible and cost-effective technological aid to traditional care.

## Methods

### Overview

Given the complex and broad nature of our research question, our results are presented in alignment with the scoping review methodology, focusing on the state of research and identification of key concepts and gaps [19,20]. Our methods are structured according to PRISMA (Preferred Reporting Items for Systematic Reviews and Meta-Analyses) principles endorsed by the Cochrane Collaboration [21] and the scoping review reporting framework [22].

### Inclusion Criteria

The included studies were published in English, examined the use of digital health tools in populations with SSDs, and published between 2010 and 2023 (given the evolution of technology during this period). Population, Intervention, Comparison, and Outcome principles guided the formulation of the inclusion criteria using the categories population, intervention, and outcome of interest (control type was not applicable as we included a range of study types; refer to Textbox 1 for details). Studies were excluded if no full text was available (eg, editorials and conference presentations), if not published in English, if examined in a population with health challenges other than SSDs (eg, depression, substance use, and physical health conditions), if they did not use technology to deliver an intervention, or if they did not examine clinical targets related to SSDs. We also did not include studies that examined the pivot to virtual care (eg, phone, video, or other communication technology-based care) during the COVID-19 pandemic and virtually delivered care more broadly, as the only technological component was videoconferencing or telephone delivery of treatment as usual (TAU). The included studies sought to enhance the delivery of TAU (ie, technologically supported TAU) or deliver adjunct interventions (ie, interventions not included in TAU).

**Study inclusion criteria following the Population, Intervention, Comparison, and Outcome principles.**

**Population diagnosis**

- Schizophrenia
- Schizophreniform
- Schizoaffective
- Psychotic disorder not otherwise specified
- Psychosis

**Type of technology**

- Mobile apps
- SMS text messaging
- Web based
- Videoconferencing
- Wearables
- Blended interventions

**Outcome target**

- Symptoms
- Functioning
- Service engagement
- Illness management

**Study methodology**

- Randomized controlled trials
- Pilot trials
- Qualitative studies
- Feasibility studies
- Protocol papers

### Search Strategy

A total of 3 core databases were searched: PsycINFO, Embase, and MEDLINE. In addition, Cochrane and PROSPERO databases were searched for existing reviews and protocols. References of resulting included studies were hand searched.

Search strategies used for general databases were as follows:

- Population: Psychosis OR Schizophrenia OR Schizoaffective OR Schizophrenia Spectrum OR Psychotic Disorders OR First-Episode Psychosis OR Early-Episode Psychosis
- Intervention: SMS OR Short Message Service OR SMS-Survey OR Texting OR Text Message OR SMS Based System OR SMS Reminder OR Text Message Reminder OR Digital Health OR Telehealth OR Mobile Apps OR Mobile Applications OR Mobile Health OR eHealth OR mHealth OR Wearable Technology

Search strategies used for databases powered by OVID were as follows: schizophrenia spectrum.mp. OR psychotic disorder.mp. OR exp psychosis/AND (sms or short messag* service* or texting or text messag*).mp. OR (mobile apps* or smartphone app* or telehealth) OR (eHealth or mHealth or mobile health or internet intervention or web-based treatment or web-based intervention or wearabl*).mp.

### Reviewer Protocol

Studies resulting from the initial search were exported into a reference manager (EndNote; Clarivate) [23], where the initial deduplication occurred, and then transferred into a web-based review management system, where the system again identified duplicates (Covidence; Veritas Health Innovation) [24]. This platform also allowed for the independent review of each study for inclusion. Authors (JD, MI, LT, AZ, and TA) conducted the review using the title, abstract, and full text. Review conflicts were reviewed by the first author (JD) and team and, if needed, by the last authors (SK and GF). At least 2 reviewers reviewed each publication.

### Data Extraction

Both qualitative and quantitative data were extracted from the included studies. First, methodological information was collected, including research design and publication type, sample size, diagnostic group, length of study, and outcome measures. Second, intervention information was collected on the digital intervention target, the type of technology used, the evidence-based approach used, and the length of the intervention. Third, study outcome data were extracted, including qualitative or descriptive and quantitative data regarding primary outcomes. Both qualitative and quantitative data are reported descriptively.

### Analysis

The reported results are descriptive, in line with scoping review methods, and provide a high-level overview of key findings. Extracted data are charted according to the type of technology used, followed by subcategories regarding the stage of research (eg, development, pilot feasibility and efficacy, and effectiveness and implementation trials), themes, and issues uncovered through data synthesis. Of note, we distinguish between blended care and multitechnological interventions. Blended care is defined as integrating technology into traditionally delivered care (ie, in-person care) [25], whereas we define multitechnology interventions as using >1 technological platform and may be integrated with traditional or virtual care. Furthermore, given the large number of included studies, findings report on primary outcomes. A more detailed review would be required to investigate secondary and tertiary findings. Primary outcomes pertaining to feasibility use highly varied measures; therefore, we provided a high-level description of outcomes.

## Results

### Overview

**Figure 1:**
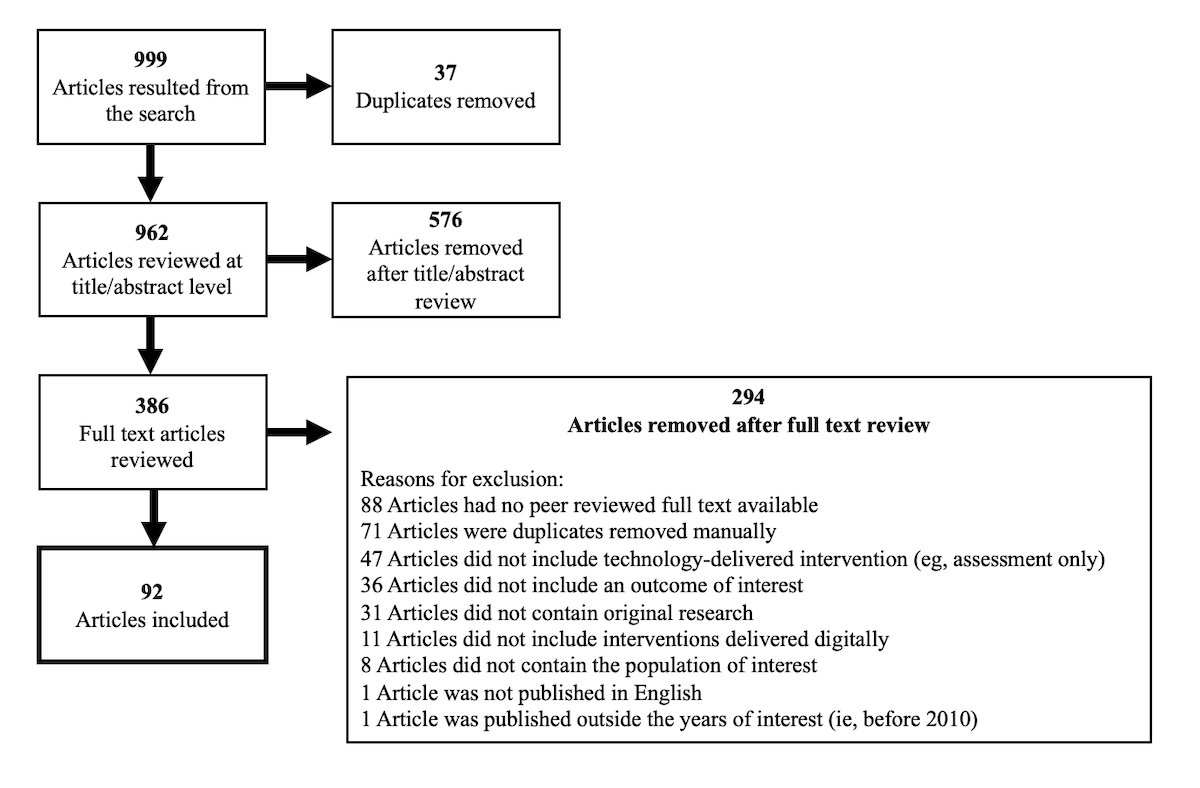
PRISMA (Preferred Reporting Items for Systematic Reviews and Meta-Analyses) breakdown of search results. Description of the review process that determined the included and excluded studies.

**Figure 2:**
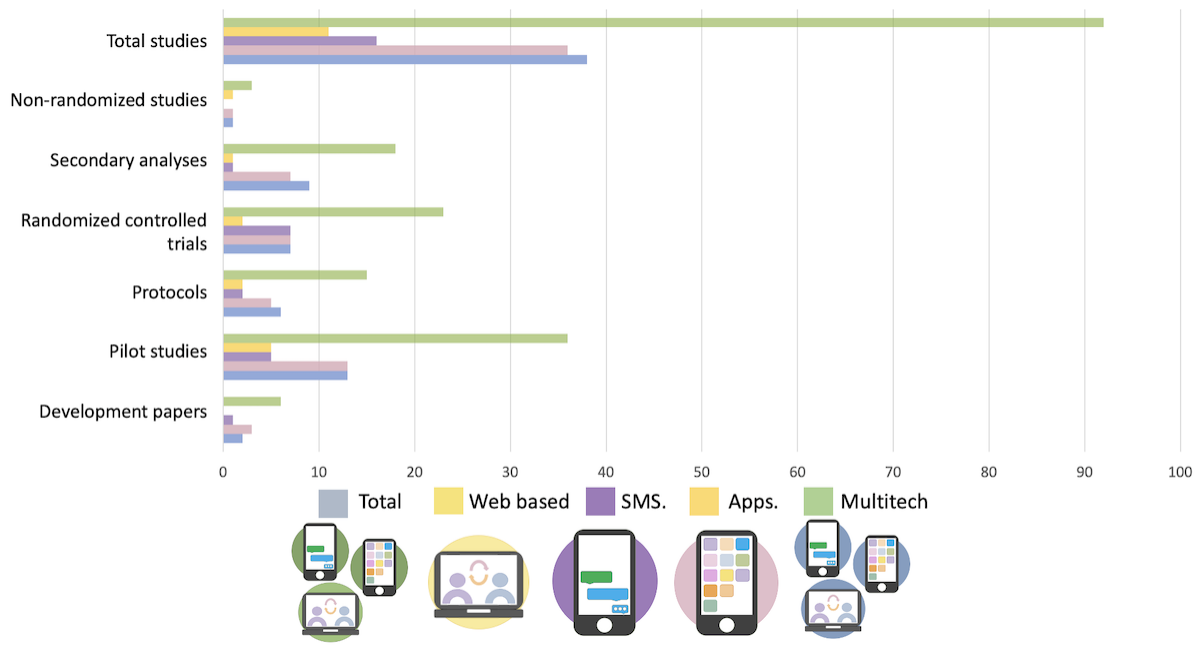
Number of publications over time by technological modality.

Our search yielded 999 studies, of which, through careful review, 92 (9.21%) studies were included (refer to Figure 1 for details of the search exclusion), investigating approximately 50 unique interventions. It is difficult to know the exact number of unique interventions as this is not always clearly reported. Included studies were published in a range of countries, predominately in North America (40/92, 43%), Europe (33/92, 36%), Australia (13/92, 14%), and East Asia (7/92, 7%). Studies were published from 2010 to 2023, with the majority published between 2018 and 2020 (50/92, 54%; Figure 2).

We primarily examined results through the intersecting lens of technology type and stage of research (ie, study design and aim). Among the 92 included studies, 11 (12%) focused on web or internet-based interventions, 25 (27%) examined smartphone apps, 16 (17%) investigated SMS text messaging, and 40 (43%) used multiple types of technology to deliver the interventions. No included studies used wearable technology to deliver an intervention. Studies included in this review that used videoconferencing or phone calls did so in combination with other types of technologies and are thus counted under multitechnology interventions.

Regarding the stage of the included research, of 92 studies, there are 5 (5%) qualitative studies investigating development and patient experiences, 32 (35%) pilot trials (26/32, 81% open trials and 6/32, 19% randomized control trials [RCTs]), 13 (14%) protocol papers, 23 (25%) conducted RCTs, 16 (17%) secondary analyses (both quantitative and qualitative), 3 (3%) nonrandomized trials, and no implementation trials. Given the number of secondary analyses, it is important to note the number of unique technologies being studied to reflect a potentially more accurate picture of the spread of digital mental health that is leveraging personal technologies in this population. There are 50 unique technologies studied within the 92 included studies. Several unique technologies (19/50, 38%) are investigated in >1 publication, with several published protocols suggesting ongoing investigations using these interventions. Contextually, it is important to note that all included trials recruited from mental health care clinics and institutions providing traditional care, and as such, all interventions included in this study can be considered blended or integrated to varying degrees with traditional care.

### Overall Feasibility

Factors of feasibility in included studies vary, such as measures of acceptability, usability, technology engagement, and user feedback. The vast majority of included studies report high levels of acceptability and usability. Technology and user feedback are mixed and reveal several high-level themes. First, technology engagement tends to decrease over time regardless of technology type. Second, many studies report self-reflection and therapeutic rapport as positive features of using these technologies. Third, various types of prompts were used with varying success to enhance engagement.

### Safety and Privacy Outcomes

Across all technologies and all study designs, 60% (55/92) of the studies did not report on any safety indices at all. The most common indicator (31/92, 34%) of safety used was reporting serious adverse events (SAE) and adverse events (AE). Most studies reported that no SAEs or AEs were related to the technological intervention, and if there were any present, they provided a short description of the SAE or AE. Furthermore, the vast majority of the studies (64/92, 70%) did not comment on the privacy measures taken by the study team or built into the technology used to deliver the intervention.

### Overall Efficacy

There were 16 RCTs and 1 pilot RCT examining the efficacy of the technological intervention as its primary outcome; see Table 1). Regarding methodology, most RCTs use TAU (10/17, 58%) as their comparison group, with others using varying active comparisons, including phone calls, films, sham apps, and other therapies. RCTs were most commonly reported using single-blind designs (7/17, 41%); however, 2 (12%) were double blinded, and 8 (47%) did not use blinding procedures for their assessors or participants. Approximately 52% (9/17) of the trials showed a positive change (ie, improvement in target) during the course of the intervention in favor of the technology, with another showing positive change in 1 primary outcome but not both [26] and another showing no overall change but a change in the positive symptom subscale scores [27]. Compared to the control, 47% (8/17) of the RCTs show significant group differences in their primary outcome, with the same 2 studies reporting significant group differences in part of their primary outcomes [26,27]. Only 4 studies reported effect sizes, with 3 being interpretable as medium to high and 1 as low [28-31]. The type of effect sizes reported were inconsistent across studies and thus were interpreted as low, moderate, and high based on suggested interpretation guidelines for each type of effect size to aid in comparison. A more detailed examination of efficacy, which includes all study types with primary efficacy outcomes, can be found in the sections below divided by technology type.

**Table 1:** Included original randomized control trials with primary efficacy outcomes.

Author, year	Technological intervention information						Study design information					Study outcomes			
	Type	Name	Target	Approach	Length (months)	Population		Sample size	Control	Blinding	Primary outcome		Significant change	Significant group difference	Effect size
Schlosser et al [32], 2018	App^a^ and SMS text message + hone	PRIME	Motivation	Mixed	3	SSDs^b^		32	TAU^c^	Single	Motivated behavior		No	No	NR^d^
Fisher et al [28], 2023	App and videoconference	PRIME	Motivation	Mixed and CR^e^	4	SSDs		100	Sham	Double	Cognitive functioning		Yes	Yes	Medium to high
Garety et al [33], 2021	App and web-assisted therapy (adjunct to 8 in-person sessions)	SlowMo	Paranoid ideation	CBT^f^	3	SSDs		361	TAU	Single	Paranoia		Yes	Yes	NR
Krzystanek et al [34], 2019	App, provider web portal, and telemedicine	MONEO	Symptom severity	Symptom monitoring and CR	12	Schizophrenia		290	Sham	None	Symptom severity		Yes	Yes	NR
Schulze et al [35], 2019	SMS text messaging and phone calls	Telca	Medication adherence	NR	6	Schizophrenia or BD^g^		120	TAU	None	Medication adherence		Yes	Yes	NR
Stentzel et al [36], 2021	SMS text messaging and phone calls	Telca	Medication adherence	NR	6	SMI^h^		118	TAU	None	Quality of Life		No	No	NR
Depp et al [29], 2019	App and single in-person therapy session	CBT2Go	Symptom severity	CBT	3	SSDs or BD1		229	TAU and sham	Single	Symptom severity		Yes	Yes	Low
Zhu et al [30], 2020	Embedded onto social media app	WeChat	Medication and illness management	NR	6	Schizophrenia		84	Phone calls	Single	Medication adherence		Yes	Yes	High
Beebe et al [26], 2014	SMS text messaging versus phone Calls	TIPS	Medication adherence and symptom severity	NR	3	SSDs		28	SMS text messaging vs phone vs combined	Single	Medication adherence and symptoms severity		Adherence: no; symptoms: yes	Adherence: no; symptoms: yes	NR
Cullen et al [27], 2020	SMS text messaging	T4RP	Relapse prevention	NR	6	Schizophrenia or SZA^i^		40	TAU	None	Symptom severity		Yes: positive symptoms only	Yes: positive symptoms only	NR
Montes et al [37], 2012	SMS text messaging	NR	Medication adherence	NR	3	Schizophrenia		254	TAU	None	Medication Adherence		Yes	Yes	NR
Pijnenborg et al [38], 2010	SMS text messaging	NR	Activity achievement	NR	1.75	SSDs		62	TAU	None	Goal attainment		No	NR	NR
Välimäki et al [39], 2017	SMS text messaging	NR	Relapse prevention	SDT^j^	12	SSDs		1139	TAU	None	Rehospitalization		NR	No	NR
Xu et al [31], 2019	SMS text messaging	LEAN	Medication adherence	NR	6	Schizophrenia		278	TAU	Single	Medication Adherence		Yes	Yes	Medium
Kaplan et al [40], 2011	Internet based	NR	Symptom severity	Peer support	12	Psychosis		300	Three conditions	None	Symptom severity		No	No	NR
Westermann et al [41], 2020	Internet based	iCBTp	Symptom severity	CBT	2	SSDs		101	TAU	Single	Symptom severity		No	No	NR
Dabit et al [42], 2021	App, telemedicine, and SMS text messaging	CLIMB	Social functioning	SCT^k^	2.25	Schizophrenia or SZA		24	Sham	Double	Social functioning		Yes	No	NR

^a^App: smartphone app.

^b^SSD: schizophrenia spectrum disorder.

^c^TAU: treatment as usual.

^d^NR: not reported.

^e^CR: cognitive remediation.

^f^CBT: cognitive behavioral therapy.

^g^BD: bipolar disorder.

^h^SMI: serious mental illness.

^i^SZA: schizoaffective disorder.

^j^SDT: self-determination theory.

^k^SCT: social cognition training.

Detailed results are outlined in the subsequent sections and focus on the stage of research and notable results. Key gaps and future directions are explored in the *Discussion* section. A summary of the included studies can be found in Multimedia Appendix 1 [26-120].

### Multiple Technology Interventions

#### Overview

The largest proportion (40/92, 43%) of the included studies investigated interventions that leveraged multiple types of personal technologies. Multiple distinct personal technologies within 1 intervention were identified if they served (1) distinct purposes or (2) two distinct modes of communication. For example, an ecological momentary assessment (EMA) embedded with a mobile app would be considered 2 distinct technologies as an EMA collects information, whereas the mobile app shares information. In contrast, an addition of modules to an already existing web platform serves the same purpose as the original web platform and would count as 1 technology. Most commonly, interventions combined the use of a smartphone app and a web page that could be accessed by either a clinician or peer support [29,33,34,43-49], with some additionally combined with targeted in-person intervention sessions [33,47]. Other technology combinations included smartphone apps to deliver resources with added EMA and intervention approaches [50] and a web page [51] or wearable technology [52] to help users self-manage their mental health. Other combinations still included using a smartphone app or web page in combination with communication technology such as SMS text messaging [29,53,54], phone calls, videoconferencing [32], and email [55]. Finally, 2 interventions engage various communication strategies, such as SMS text messaging and phone calls [35,56] and a smartphone app, SMS text messaging, and telemedicine [42].

Of the 40 included studies investigating interventions leveraging multiple personal technologies 2 (5%) were development papers [57,58], 14 (35%) were pilot studies [42,43,45,49,51,53,54,59-65], 6 (15%) were RCT protocols [47,48,52,56,66,67], 8 (20%) were RCTs [28,29,33-36,64], 9 (23%) were secondary analyses [44,55,68-72,74,75], and 1 (2%) was a nonrandomized trial [46]. There are 17 unique multitechnology interventions investigated using the 40 included studies. Targets of the multitechnology interventions included illness self-management (5/17, 29%) [43,52,55,58,62], relapse prevention (3/17, 18%) [46,47,49], medication adherence (2/17, 12%) [51,56], general symptom severity (2/17, 12%) [29,34,48], social functioning (1/17, 6%) [42], motivation (1/17, 6%) [53], coping with auditory hallucinations (1/17, 6%) [50], negative symptoms (1/17, 6%) [54], and cognitive function (1/17, 6%) [70]. Evidence-based approaches are reported by 77% (13/17) of these interventions, including cognitive behavioral therapy (CBT; n=3, 23%); peer support (n=1, 8%); cognitive remediation (n=1, 8%); information motivation behavioral skills (n=1, 8%); connectedness, hope and optimism, identity, meaning, and empowerment (n=1, 8%); social cognitive theory (n=1, 8%); and mixed approaches (n=6, 46%). For all studies (29/92, 32%) that investigated any of the 17 multitechnology intervention, the trial lengths ranged from 1 visit to 60 months, with a mode of 3 months.

#### Feasibility

Studies (23/40, 58%) investigating feasibility-related variables as the primary outcome reported varying measures of engagement, including study retention, intervention retention, and various means of measuring technology engagement (eg, number of log-ins per period, number of tasks completed, and number of responses). Regarding study retention, included studies reported good retention rates of 78% to 100% [28,43]. However, technology or intervention retention tended to decrease over time and was 42% to 52% at the end of the intervention periods [43,60]. Technology use over time also tended to decrease over time regardless of the measurement [28,43,60]. Despite this, studies reported high levels of response or completion throughout the study period (70%-84%) [49,51,53,59]. One study reported a relatively low response rate (22%); however, this represented a homework completion rate of at least once per week [54]. Another study reported on clinician engagement with the connected clinician portal (67% log-in rate), which was much lower than patient engagement (80% response rate) [49]. Some studies reported on factors impacting intervention engagement, which included low mood or depression [44,68], negative symptoms and low motivation [44], and fluctuations in wellness [71]. Technological factors were also reported to impact engagement. Specifically, ease of navigation, accessibility of resources, the fit of technology [44,71], and concerns regarding privacy [69] impacted engagement, whereas coaching facilitated engagement [53,55] as long as it did not feel too scripted [53]. Overall, patients tended to endorse the technologies as easy to use and helpful and were satisfied with the technology or interested in continued use [43,49,51,53,117].

Of note, 7 (30%) feasibility studies of the 23 included multitechnology studies focused on 1 intervention [58,61,63-65,72,75] in varying populations (ie, psychotic and mood disorders), in different contexts (ie, outpatient, assertive community treatment, and veteran association hospital), and with different comparison groups and augmentations (ie, the addition of video content and phone calls from interventionists). Throughout these studies, participants and clinicians rated the acceptability and usability of the intervention as high. Participants could access the app at any time, and the app sent out prompts and phone calls to support app engagement. Participants responded to most prompts [64,65] and phone calls from the mobile case manager or interventionist [65]. Despite this, patient app engagement over time declined in the studies that measured use over time [63,64] but reported better engagement over time compared to an in-person group [64]. One study reported on cost-effectiveness and found that the technologically supported intervention was significantly less costly, with no significant differences in efficacy compared to a fully clinician-delivered intervention [73].

#### Efficacy

Studies examining the efficacy of multitechnology interventions as a primary outcome (10/40, 25%) [29,32-36,42,46,70,74] reported mixed findings across a wide range of specific outcomes. One study examining self-guided CBT did not report an overall significant difference in symptom severity compared to TAU [29]. Another study investigating technology-assisted, clinician-delivered CBT for paranoid thinking found that the intervention significantly reduced paranoia across 3 measures at the end of a 3-month intervention compared to TAU [33]. Studies investigating social-cognitive-functional domains reported significant impacts on aspects of global cognition, attention [28], motivated behaviors (ie, anticipatory pleasure and improved effort expenditure for future social interaction) [32], improvements in affective symptoms [34], the identification of correct answers and cognitive fatigue [70], and social functioning [42]. A smartphone app–clinician portal alert system reported significant reductions in relapse, rates of hospitalization, and urgent care needs [46]. An intervention comprising biweekly clinician phone calls and weekly SMS text message check-ins showed improvements in medication adherence compared to controls [36]; however, it did not show improvements in other areas such as global functioning or quality of life [35].

### Smartphone App Interventions

#### Overview

Of the 92 included studies, research investigating the use of smartphone apps included 25 (27%) publications, of which 2 (8%) were development papers [76,77], 8 (32%) were pilot studies [76,78-84], 1 (4%) was a nonrandomized study [85], 6 (24%) were protocol papers for RCTs [50,86-90], 3 (12%) were RCTs [30,91,92], and 5 (20%) were secondary analyses [93-97]. The average length of interventions was 4 months (range: 1 visit to 12 months and modes: 3 and 12 months). Pilot feasibility studies typically lasted 1 to 2 months; pilot efficacy trials lasted 2 to 6 months; and randomized trials lasted 3 or 12 months, including protocols. Sample size within trials is also highly variable (mean 41.65; median 31, range 8-229).

There were 15 unique smartphone app interventions investigated in the 25 included studies. Most of the included smartphone interventions were positioned as adjunctive to traditional mental health care or TAU and were aimed at illness self-management [76,80,88,91]; medication adherence [30]; and symptom severity [81,86,89,92], including specific domains such as auditory hallucinations [84,90], social skills [79,82], and metacognition [85]. Most apps reported evidence-based foundation therapeutic approaches, most commonly CBT (6/15, 40%) [76,78,81,83,84,89], followed by social skills training (1/15, 7%) [77], positive psychology (1/15, 7%) [82], acceptance and commitment therapy (1/15, 7%) [76], metacognitive intervention for schizophrenia (1/15, 7%) [85], or report mixed approaches (3/15, 20%) [88,91]. However, some (2/15, 13%) [30,80,90] interventions do not report following a specific evidence-based approach or omit this information. Two (2%) of the 15 included app interventions were specifically blended with in-person care [84,92].

#### Feasibility

Of the 25 studies investigating apps, there were 12 (48%) studies that reported primary outcomes related to the feasibility of apps [78,80-84,91-96]. All feasibility studies investigating apps reported high levels of acceptability, usability, and satisfaction. Studies using a prompt or EMA system reported medium to high response rates of 33% to 81% [33,92,93]. Studies reporting on task or module completion also reported medium to high completion rates of 42% to 95% [78,81,82,92]. Of 12 feasibility studies, 2 (17%) studies reported that engagement with apps decreased over time [91,94], 1 of which reported that 50% stopped using the app within 3 months [91]. Facilitators of app engagement were functions allowing synchronous communication with clinicians [81], integration of face-to-face sessions [96], and stronger therapeutic alliance [94], whereas barriers to engagement were lack of clinician support using the app, concerns regarding therapy [91], feeling the app was not personable, and there were too many sessions or prompts [81,83]. Some studies found participant factors associated with app engagement, such as age, employment status, race, and smartphone ownership [93,94].

#### Efficacy

Of 25 studies, 4 (16%) examined the efficacy of interventions delivered via smartphone apps [30,79,85,98], of which study examined an app delivering self-guided CBT [98]. Regarding overall symptom severity, a pilot study found significant changes across recovery and symptom outcomes [98]. Another pilot study examined the delivery of self-guided social skills training and found a moderate impact on social functioning at the end of the intervention; however, it found that gains were not maintained at follow-up [97]. A nonrandomized control comparison study found that a smartphone-delivered metacognitive therapy augmented by weekly mentoring sessions found positive impacts on metacognition, delusions, general pathology, and negative symptoms [85]. Finally, an RCT examined the effect of medication reminders sent through a smartphone app and reported that compared to TAU, those receiving reminders showed significantly better medication adherence, with large effect sizes reported [30].

### SMS Text Messaging Interventions

#### Overview

In this review of 92 studies, 16 (17%) were included in which an intervention using SMS text messaging was investigated. Of the 16 papers, 1 (6%) was a development paper [99], 2 (12%) were protocol papers [100,118], 4 (25%) were pilot trials (ie, 2 (12%) were open trials [101,102] and 2 (12%) were pilot RCTs [103,104]), 6 (38%) were RCTs [26,27,31,37-39], and 3 (19%) were secondary analyses [105-107]. The sample sizes varied greatly between 14 and 1139 (mean 181, SD 274.7); however, most were <100 participants (10/16, 62%). The length of the intervention varied from 1 to 18 months, with a mean of 4 months and a mode of 6 months.

There were 11 unique SMS interventions investigated within the included 16 studies. Most (n=9, 82%) did not report following a specific evidence-based model, with one reporting the use of CBT [102], and another Self-Determination Theory [39]. Interventions using SMS text messaging targeted a variety of outcomes, mainly in the form of reminders, such as medication adherence (4/16, 25%) [26,31,37,102], appointment attendance (1/16, 6%) [100], and relapse and rehospitalization rates (1/16, 6%) [27]. Beyond service engagement, some studies aimed to support clinical outcomes, such as auditory hallucinations [102], social skills [102], goal attainment [38], and general support [39,101,103,105]. Most SMS-based interventions were framed as adjunctive care, and only 1 was reported as being intentionally blended with traditional care [103]. Most SMS-based interventions did not report to follow a specific evidence-based approach. One intervention was based on CBT [102], and another was based on self-determination theory [39].

#### Feasibility

Of the 16 studies investigating SMS text messaging, 5 (31%) reported feasibility as the primary outcome. Studies reported high text message response rates (69% to 87%) [101,103,104] and high levels of satisfaction, ease of use, and helpfulness of SMS text messages [103,104,107]. One of the 5 feasibility studies even found that therapeutic alliance was higher with the SMS text message interventionists compared to participants’ community-based clinicians [101]. Another study explored areas that participants found useful to discuss with SMS text message interventionists, including mental health symptoms, coping strategies, lifestyle or well-being, social or leisure activity, motivation, and independent living skills [106].

#### Efficacy

Of the 16 included studies that investigated SMS text messaging, 13 (81%) reported on efficacy outcomes. The reported efficacy of SMS text messaging interventions seemed to vary depending on the outcomes measured. Most studies investigating SMS interventions aimed at medication adherence were associated with greater rates of adherence [31,37,102,105], especially among individuals with schizophrenia who were living independently [102]. Effects on medication adherence may be bolstered by the addition of phone check-ins by clinicians [26]. However, drop-off effects may occur once the reminders are no longer delivered [119]. In addition, there seemed to be potential positive effects on rehospitalization rates [31]; however, results were mixed as other researchers did not find evidence of a reduction in hospital admission rates, the time between hospitalizations, or time spent in a psychiatric hospital [39]. Regarding changes in recovery and symptom severity, some studies reported improvements in negative symptoms, positive symptoms [27], cognition, and global clinical symptoms [37]. These results were strengthened when the SMS text messaging intervention was coupled with a secondary intervention (eg, telephone intervention or assertive community treatment) [26,103]. Other researchers did not find significant improvements in symptom severity [39]. Mixed outcomes were reported for social and community functioning [38,104,105]. Studies reported mixed findings regarding service engagement. One study reported improved therapeutic alliance with their mobile clinician compared to in-person clinician visits, and participant feedback results reflected a sense of support among users when receiving daily SMS text messages [101,106].

### Internet-Based Interventions

#### Overview

Of the 92 included studies, 11 (12%) described internet-based interventions that were not paired with other technology. Of these 11 papers, 1 (9%) was a cross-sectional study [108], 5 (45%) were pilot trials [109-112,120], 2 (18%) were RCT protocols [113,114], 2 (18%) were RCTs [40,41], and 1 (9%) was a secondary analysis [115]. The sample size of included studies examining internet-based interventions ranged from 10 to 300, with approximately 50% being >100 (mean 97). The length of the intervention ranged from 2 to 26 (mean 9; modes 2 and 18) months. There were 8 unique interventions investigated within the included 11 studies. The internet-based intervention treatment targets included recovery [110,113], cognition [112,120], working memory [111], symptom severity [40,41], and illness self-management [108]. Most internet-based interventions reported to be based on an evidence-based framework, including connectedness, hope and optimism, identity, meaning, and empowerment [110]; cognitive remediation [111,112,120]; CBT [41]; peer support [40]; and mixed approaches [113].

#### Feasibility

Of the 11 studies examining internet-based interventions, 6 studies examined feasibility using varied measures of feasibility and engagement. Study attrition was reported between 64% and 100% [109,112,114,120]. Given the range of interventions delivered, intervention engagement was measured differently in each study based on the intervention design. Of the 11 studies, 1 (9%) reported the number of log-ins (eg, mean 39.2 over the intervention) [109], 1 (9%) reported the mean time spent on the website per week (eg, 3 h/wk) [120], another (9%) reported percentage of participants completing at least 80% of modules according to a predetermined meaningful completion rate (eg, 70% of participants completed 80% of modules) [112], and 1 (9%) reported the percentage of participants that used the website (eg, 41% of participants used the website) [108]. Of the 11 studies, 2 (18%) reported that participant factors impacted engagement, such that younger age [108,120], higher education [108], being employed [108], and lower cognitive symptoms [120] led to better engagement.

One study specifically reported integrating a website into regular therapy sessions and found that the website was used in 95% of therapy sessions [114]. Qualitative feedback related to the use in sessions included that the website helped facilitate conversation and reflection in their sessions and was a key tool for engagement, leading to increased perceived recovery [114]. However, only 60% of participants reported use outside of sessions [114]. Another study presented findings related to a self-guided website [115] and found that the self-guided nature to some was motivating and cultivated a sense of autonomy in recovery, whereas others found the self-guided nature overwhelming, which interrupted engagement. Positive aspects reported related to the self-guided website were having at-demand resources more so than any clinician-driven service, having the means to distract from distress in a meaningful, positive way, and having the ability to interact with peers and psychoeducation was a normalizing experience [115].

#### Efficacy

Of the 11 studies examining internet-based interventions, there were 3 (27%) that reported primary outcomes pertaining to the efficacy of internet-based interventions [40,41,111]. Each of these interventions focused on different evidence-based approaches. One study examined the impact of online peer support (ie, comparing anonymous listserve group email communication or communication with peers via web-based bulletin board compared to TAU) on subjective recovery, quality of life, empowerment, and social support [40]. No significant group differences were found with regard to online peer support and the control group for either the listserve or bulletin board communication types [40]. Another study investigated remote cognitive remediation (ie, web-based computerized tasks, psychoeducation, and strategy development paired with weekly clinician calls) targeting working memory [111]. Compared to TAU, remote cognitive remediation was associated with improved working and episodic memory, with medium effect sizes reported [111]. Finally, another study looked at the effects of self-guided CBT for psychosis on various psychotic symptoms compared to a waitlist condition [41]. Individuals receiving self-guided CBT for psychosis demonstrated greater improvements in symptoms overall and self-reported hallucinations compared to the waitlist condition, with a small to medium effect size reported [41]. However, the study did not find significant differences in other positive psychotic symptoms [41].

## Discussion

### Principal Findings

The primary aim of this review was to provide a high-level overview of the use of personal technologies in research on digital mental health interventions for individuals with SSDs. Publication patterns show rapid growth in the area over the past 5 to 7 years, highlighting increasing interest in this area [10-12]. The lower number of publications reported for 2021 to 2023 is likely partly attributable to a combination of the large number of protocols published in 2020, as many of these trials may still be underway, and research challenges related to the COVID-19 pandemic. Personal technologies leveraged most often included the use of smartphone apps or an app in combination with other technology, such as a clinician web portal.

Our review confirms that personal technologies have already garnered acceptance among individuals with SSDs, as evidenced by consistently high ratings of acceptability, usability, and satisfaction. Some studies even report that augmenting care with technology improved engagement in traditional in-person or remote care [44,101,106]. In included studies which report participant feedback, participants typically report that these technologies facilitate self-reflection and understanding, better communication with their clinical teams, improved access to evidence-based resources, and support in times of need. Findings related to feasibility presented in this review are similar to findings reported elsewhere for groups with SMI [121].

Despite the high levels of acceptance and interest, sustained engagement in technological interventions is an ongoing concern [122] and is not well understood. Measures of engagement are highly inconsistent across studies, and include module completion, response rates, skills and homework completion, and number of posts, making it almost impossible to understand a cohesive story regarding engagement with digital tools in groups with SSDs. While simple metrics such as screen clicks or the number of days or hours used are commonly reported, the field lacks an understanding of clinically meaningful engagement for this metric [123]. Beyond access metrics, there is no consistent measure of technology use. The challenge of heterogeneity in engagement metrics is already well known [124], but a lack of progress in agreement around these common metrics hinders future advances in the field [125]. Furthermore, likely in part owing to a lack of definition in this area, means of addressing technology disengagement are also poorly understood. A review examining the use of persuasive system design (design features designed to address or enhance engagement) in technologies designed for use in depression and anxiety reported that persuasive system design efforts did not systematically lead to improved engagement but are associated with improved efficacy [126].

Moreover, some ongoing challenges may impact the feasibility of using such technologies in practice, including understanding the link between privacy protections embedded in these technologies and meaningful engagement. Privacy concerns are seldom outlined in the included publications, which may result from the dearth of available guidelines, policies, and reporting standards. Privacy concerns are often cited by health care providers as a key barrier to the use of technology in practice [127], and as such, to encourage technological adoption, more data on privacy features needs to be made available. There is also a dearth of information regarding patient safety and urgent or crisis resources and support. In this review, most of the included studies (55/92, 60%) did not report on safety at all, which is well above the proportions reported across the field of digital mental health (35%) [128]. The most common safety indicators reported in this review and across the field [128] were SAEs and other AEs, which are often mandatory reporting standards for ethics boards and do not provide nuanced information regarding technology use and patient well-being.

Furthermore, there are promising early results regarding efficacy, but overall findings are mixed. Pilot studies tend to show more optimistic outcomes across a range of mental health outcomes; however, once studies reach the RCT phase, findings become more mixed. Similar descriptions are found in other areas of SMI, such as bipolar disorder [129]. In areas such as depression and anxiety, where more research on efficacy has been conducted, clearer conclusions about effectiveness can be drawn [130]. In the included RCTs, it does not appear that study procedures, such as blinding, type of technology, or sample size, systematically impact outcomes, as no obvious patterns have emerged between study type or methodology. However, with few studies featuring an active control group, any assessment of efficacy is still preliminary. Future studies should consider the active intervention when planning a control group and the purpose of the technology (eg, if testing an evidence-based treatment that is typically delivered in person and not included in TAU, then TAU would not be an active control group, such as an in-person service delivering the evidence-based treatment would better represent an active control). However, there are some interventions that have garnered significant support and exhibit positive early results [33,53,60,84,86,113].

Of note, all included studies used samples of individuals currently in treatment and thus were all offered adjunctly to traditional clinician-delivered care. However, few studies described how the technology was blended or integrated into the care structure. As such, the field provides little direction on the role of technology and its integration into existing care models. Furthermore, there were few clinical integration and effectiveness studies, underscoring the next frontier for this research in SSDs. It is important to understand how external factors, including primary treatment methods, may impact engagement, efficacy, and effectiveness. For example, the included studies that described the role of technology within the clinical context described technology-forward (ie, self-guided and clinician-supported) and clinician-forward (ie, clinician-delivered and technology-supported) interventions. Clinician-forward interventions, in our small sample of studies that described clinical integration, seemed to demonstrate better engagement [96] and better outcomes [33,84,96] than technology-forward interventions, depending on the target and treatment approach [29].

Other reviews reported mixed findings in relation to blended (technology-supported) approaches. One review and meta-analysis of digital mental health interventions across disorders suggested that technology-supported interventions outperform (Hedges *g*=0.16; *P*<.001) traditional TAU (solely clinician-delivered) [131]. However, this review did not report on the more nuanced technological differences that we highlight in this review regarding whether the intervention was delivered by the technology (ie, technology-forward) or supported clinician-delivered care (ie, clinician-forward). Another review and meta-analysis suggested that technology engagement was associated with efficacy regardless of intervention type (ie, guided or unguided) [132], suggesting that engagement may be the underlying mechanism for effectiveness in these trials rather than the level of clinician involvement. Therefore, more research is needed to define the role of technology within clinical spaces to understand the implementation and best practices related to technology-assisted care.

Future research in this promising area should focus on the identified key gaps to move the field toward implementation readiness. First, studies should clarify the technology’s intended role, intervention targets, and functionality. This is important for understanding implementation into existing clinical structures and interpreting study outcomes. Second, given the significant barriers of safety and privacy to clinical adoption, a more detailed exploration of safety and privacy indices is critical. Finally, a better understanding of barriers to participating in and disengagement from digital interventions is crucial for understanding its usefulness and scope in clinical settings.

### Limitations

There are several limitations to this review. First, as a scoping review, we could not provide a detailed review of each study but rather a high-level examination of the findings in the field to date. Therefore, data have been synthesized and presented in accordance with outlined scoping review guidelines, and, as such, the methodological quality of each study was not rigorously reviewed. Second, we limited our search to using personal technologies to deliver or support specialized care for individuals with SSDs. Other technologies are being used in SSD treatment that this review did not include, such as virtual reality, avatar therapy, cognitive remediation, brain stimulation, and more. Third, we restricted this review to outcomes directly related to general recovery (ie, core symptoms and functioning) and treatment engagement and did not focus on specific domains such as cognitive deficits. Fourth, other interventions exist for comorbid conditions, such as substance use and smoking cessation [133], physical activity [134], and resources for family and social support [135]. Finally, we only included technologies that were used to deliver interventions; we did not include studies that were used to improve the understanding of SSDs, such as observational methods (eg, technology-assisted EMA or health services–based interventions, including those that are embedded in electronic medical records for medication algorithms, symptom monitoring, or other purposes). Future reviews may consider focusing on these additional areas of digital mental health to understand how other technologies may support the recovery of individuals with SSD diagnoses.

### Conclusions

Overall, using personal technologies to deliver specialized care requires more careful consideration before advocating for broad implementation, highlighting the same challenges as other psychotherapeutic intervention research. Namely, the prolonged time between investigation and implementation is due to varied findings and a lack of cohesive targets and direction. Despite these challenges, as evidenced by this review, there is great promise for leveraging personal technology in mental health care to help provide pieces of the holistic care necessary for recovery in SSDs. Multifaceted mental health conditions such as SSDs are highly heterogeneous and may require multifaceted and flexible interventions. Although the exact nature of meaningful technological support is still being discovered, the studies in this review overwhelmingly demonstrate how flexibly personal technologies can support recovery from medication adherence to potentially delivering complex psychosocial interventions such as CBT. Furthermore, studies clearly demonstrated the feasibility of personal technologies to extend access to specialized information beyond traditional care and into everyday life. Therefore, these technologies are well suited to be integrated into existing specialty care structures for individuals with SSD diagnoses.

## Appendix

Multimedia Appendix 1 Supplemental table of included studies divided by technology type.

Multimedia Appendix 2 PRISMA-ScR (Preferred Reporting Items for Systematic Reviews and Meta-Analyses Extension for Scoping Reviews) checklist.

## Abbreviations

AE: adverse event
CBT: cognitive behavioral therapy
EMA: ecological momentary assessment
PRISMA: Preferred Reporting Items for Systematic Reviews and Meta-Analyses
RCT: randomized control trial
SAE: serious adverse event
SMI: serious mental illness
SSD: schizophrenia spectrum disorder
TAU: treatment as usual
