# Supplementary material for: Leveraging Personal Technologies in the Treatment of Schizophrenia Spectrum Disorders: Scoping Review
Source: JMIR Ment Health. 2024 Sep 30;11:e57150. doi: 10.2196/57150 (PMC11474131; doi:10.2196/57150)
Supplement: Multimedia Appendix 1 [file mental_v11i1e57150_app1.docx]

**Multi-technology Interventions**


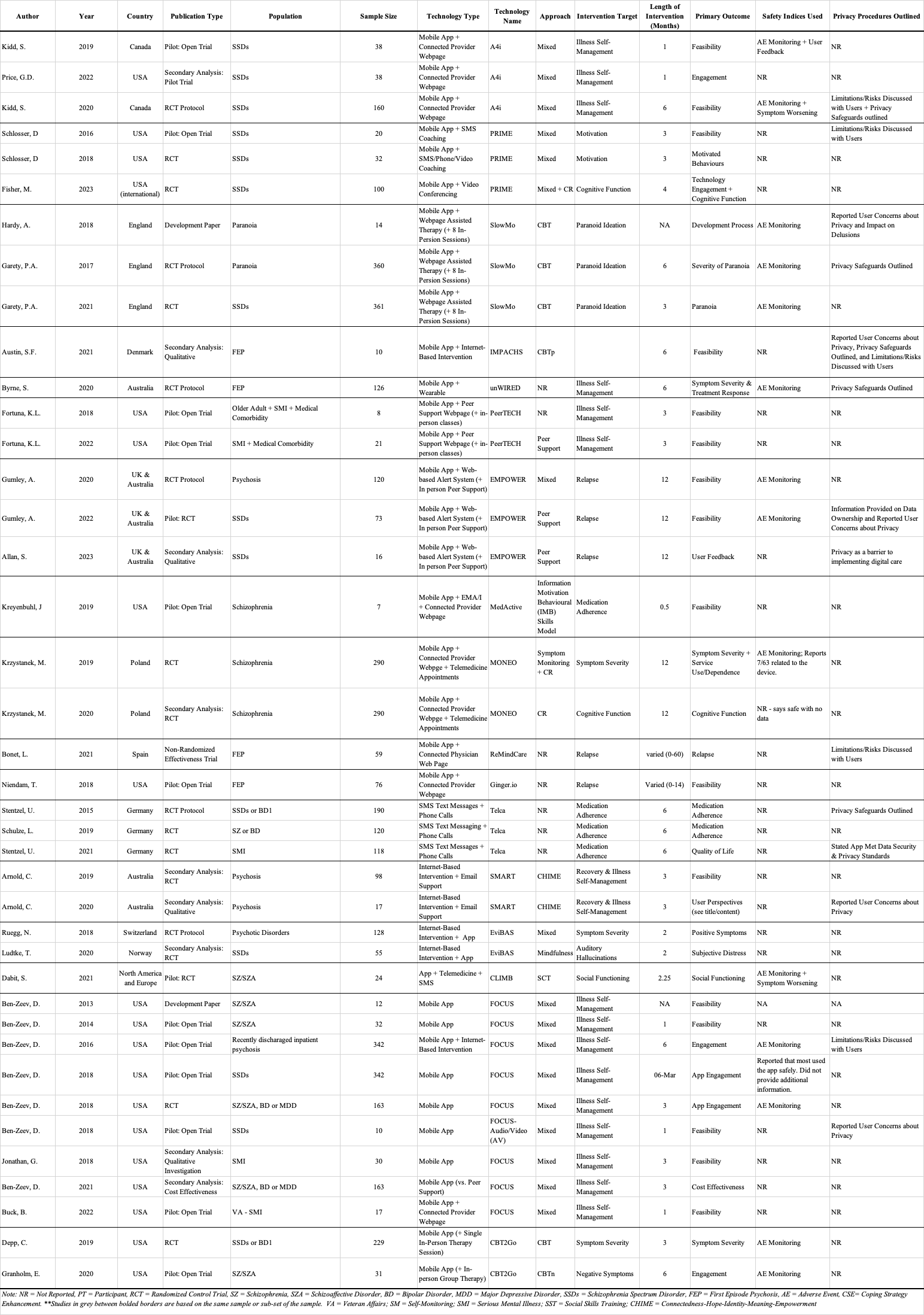


**Smartphone Applications**

**
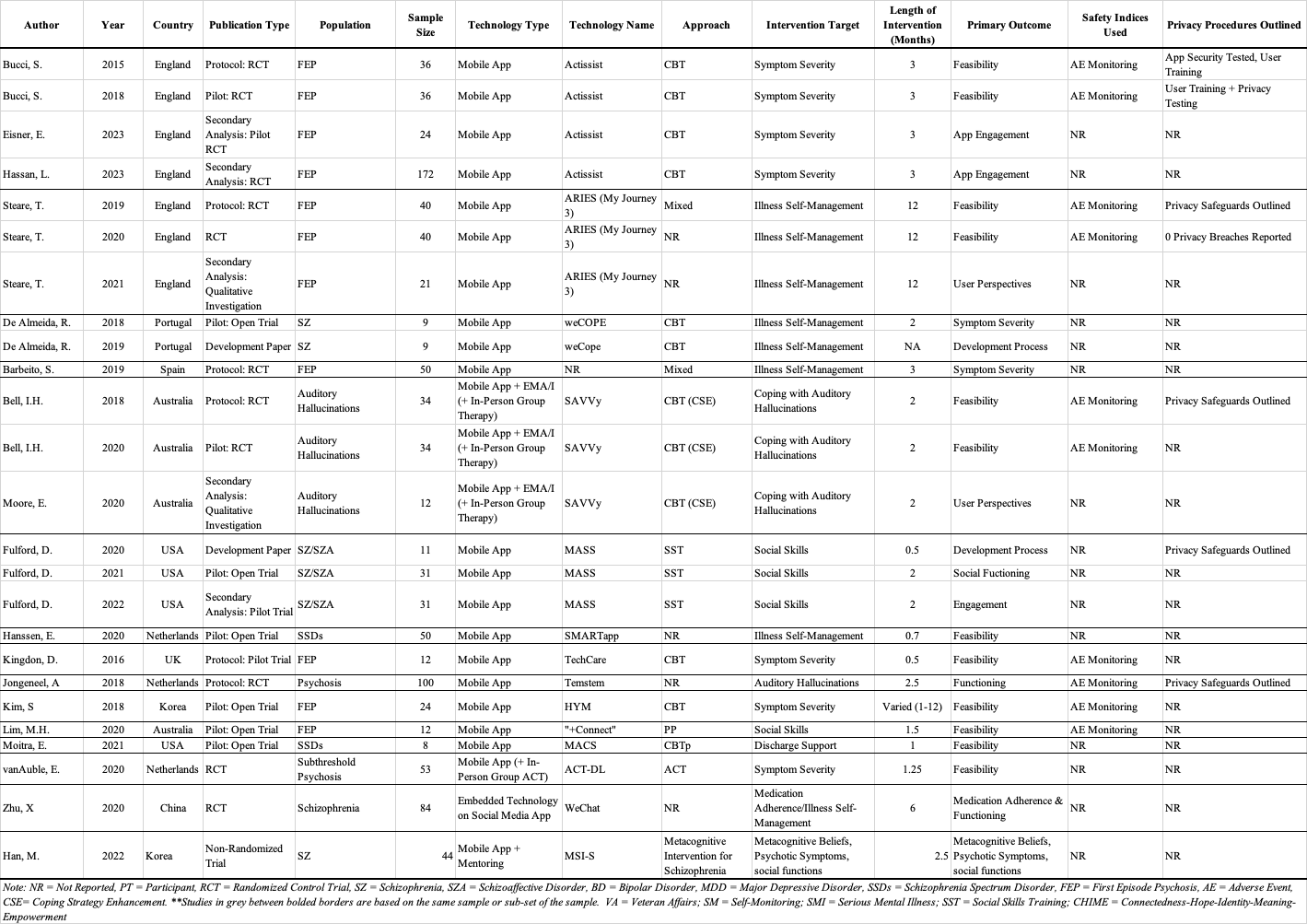
**

**SMS Interventions**


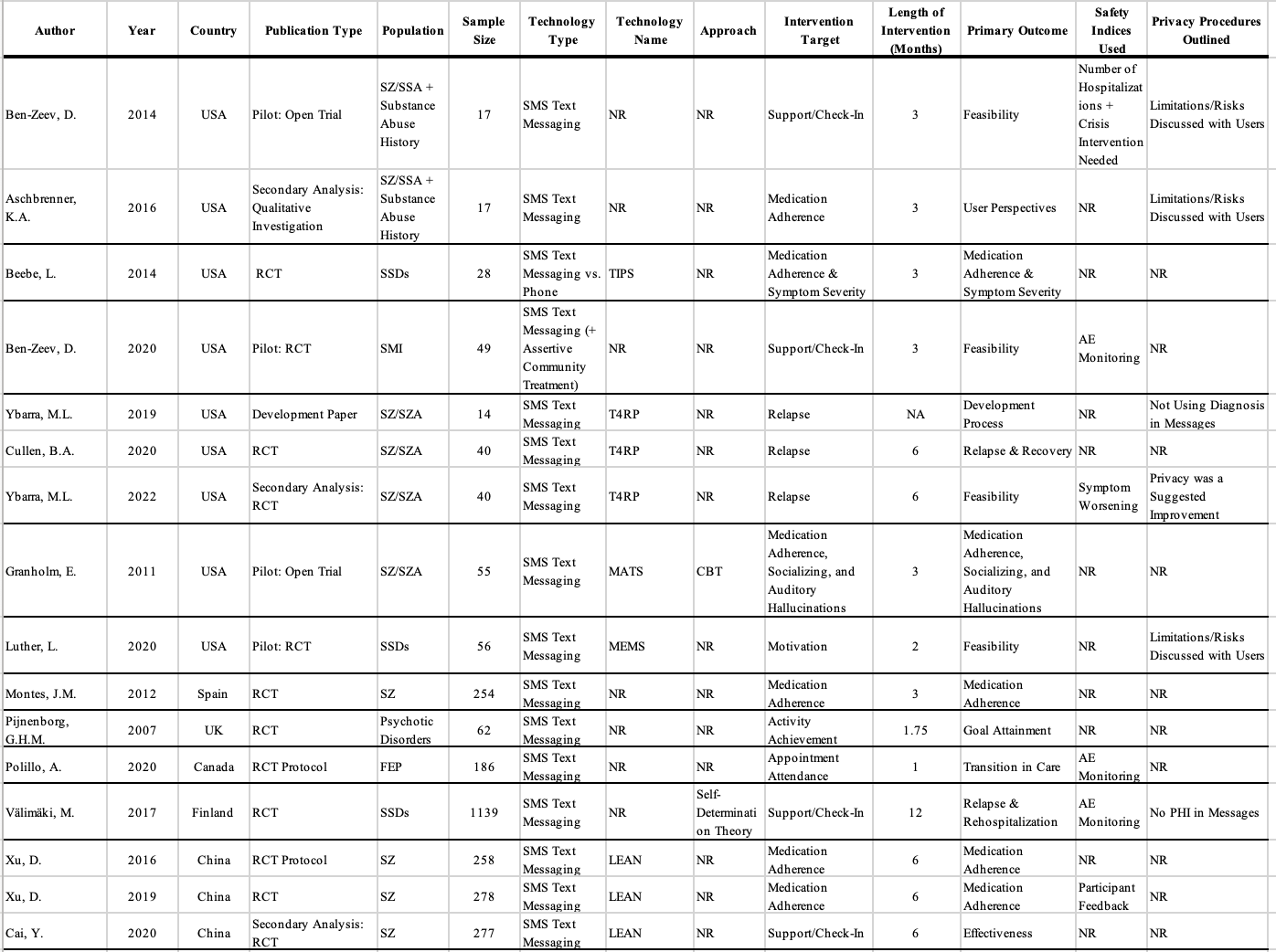


**Internet-Based Interventions**

**
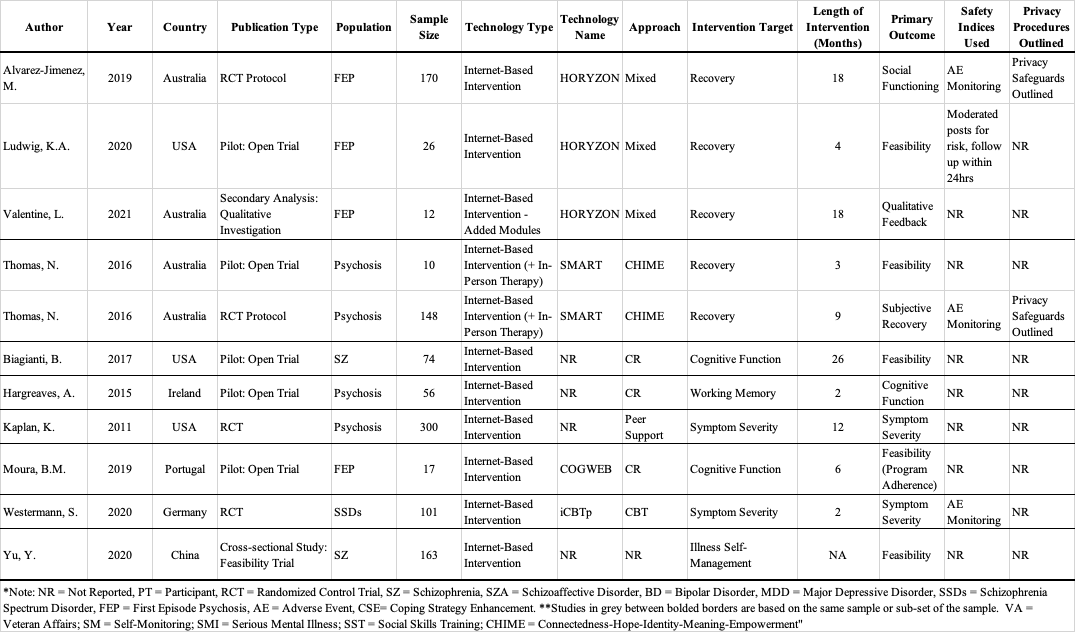
**
